# Emergency liver transplantation combined with multivisceral resection after severe abdominal trauma: a case report

**DOI:** 10.3389/fsurg.2026.1914056

**Published:** 2026-08-10

**Authors:** Marco Brolese, Matteo Pittacolo, Alessandro Vitale, Domenico Bassi, Alberto Friziero, Enrico Gringeri, Annalisa Dolcet, Francesco E. D’Amico, Riccardo Boetto, Jacopo Lanari, Alessandro Furlanetto, Alessandra Bertacco, Gianfranco Da Dalt, Renato Salvador, Alberto Brolese, Michele Valmasoni, Umberto Cillo

**Affiliations:** 1 Hepato-Pancreato-Biliary Surgery and Liver Transplantation Unit, Department of Surgery, Oncology and Gastroenterology, University of Padova, Azienda Ospedale Università Padova, Padova, Italy; 2 1st General Surgery, Acute Care Surgery Unit, Department of Surgery, Oncology and Gastroenterology, University of Padova, Azienda Ospedale Università Padova, Padova, Italy; 3 Department of General Surgery and Hepato-Pancreato-Biliary Surgery, Azienda Sanitaria Universitaria Integrata del Trentino (ASUIT), Trento, Italy

**Keywords:** case report, damage control surgery, emergency surgery, liver injury, liver transplantation, multivisceral resection, polytrauma

## Abstract

**Background:**

Liver transplantation (LT) after severe polytrauma is exceptionally rare. When damage-control surgery fails, multiorgan resection may be required as a life-saving procedure, with emergency LT representing the ultimate salvage option for patients with uncontrollable hepatic hemorrhage or irreversible liver failure. We report, to our knowledge, the first documented case of concomitant liver transplantation, splenectomy, nephrectomy, and total pancreatectomy following severe abdominal trauma.

**Case presentation:**

A 19-year-old male sustained severe blunt abdominal trauma following a motorcycle-truck collision. Imaging revealed an AAST grade V liver injury with major vascular and biliary disruption, complete avulsion of the right renal artery (AAST V), splenic injury (AAST III) and an AAST grade II duodenal hematoma. Despite damage-control surgery, followed by splenectomy and right nephrectomy, definitive hemorrhage control could not be fully achieved. Given the persistent hepatic bleeding and the expected progression to acute liver failure secondary to extensive hepatic disruption, the patient underwent emergent LT. Due to concerns for progression of the duodenal injury, a planned second-look operation was performed 48 h later, revealing marked enlargement of the hematoma with impending duodenal wall rupture, prompting the decision to proceed with total pancreatectomy. The postoperative course was uneventful, and the patient was discharged 18 days after transplantation. At 29-month follow-up, both graft function and clinical status are excellent.

**Conclusions:**

Emergency LT may represent a feasible and life-saving strategy in highly selected patients with devastating abdominal trauma. This case demonstrates that, despite the extreme surgical complexity, favorable long-term outcomes can be achieved, supporting the non-futility of transplantation in carefully selected trauma recipients.

## Introduction

Trauma represents a leading cause of morbidity and mortality worldwide, particularly among young adults and middle-aged individuals, with a large-scale U.S. study reporting a 91% increase in trauma-related mortality between 2000 and 2020 (1). Polytrauma, defined as the presence of multiple severe injuries affecting at least two body regions, is observed in approximately 51% of all trauma cases, and is commonly associated with high-energy mechanisms such as motor vehicle collisions or falls (2). As reported in the literature, in abdominal trauma, liver injury occurs in approximately 15% of patients, while concomitant involvement of two or more solid organs is observed in approximately 20% (3). The American Association for the Surgery of Trauma—Organ Injury Scale (AAST-OIS) classification is historically used to assess abdominal organs damage severity; however, this system does not directly dictate treatment strategies (4). As outlined in the latest World Society of Emergency Surgery (WSES) guidelines, non-operative management (NOM) represents the best approach when feasible, with surgical intervention reserved as a salvage strategy in case of hemodynamic instability, regardless of AAST grade (5, 6). Surgery should follow damage control surgery (DCS) principles, ensuring rapid control of bleeding and contamination to enable physiological stabilization. Severe hepatic injuries require adequate perihepatic packing, manual compression of the liver parenchyma, and the Pringle maneuver (the “Packing, Push, Pringle” rule), which represent essential techniques that every general surgeon should master. These maneuvers facilitate resuscitation and the following correction of the lethal triad - coagulopathy, hypothermia, and acidosis - in the intensive care unit (5). In trauma patients multivisceral resections are often required, whereas liver transplantation (LT) represents a rare, life-saving procedure reserved for cases of uncontrollable hemorrhage or extensive hepatic necrosis, when all other treatments have failed (7). Accurate patient selection is essential to maximize survival benefit while avoiding the waste of a scarce donor organ. A comprehensive and timely assessment of the trauma severity, patient-specific characteristics, infectious risk, and life expectancy is crucial to guide the decision-making process. We report an exceptional case of a young male patient with severe multivisceral trauma following a motor vehicle collision, requiring LT, splenectomy, right nephrectomy, and total pancreatectomy.

## Case presentation

**Figure 1:**
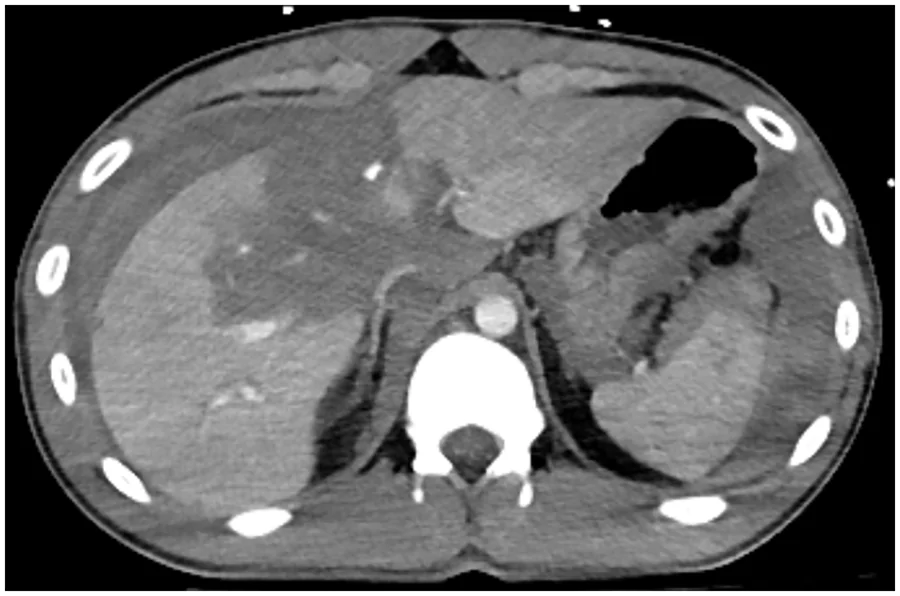
Admission CT scan demonstrating an extensive central laceration of the hepatic parenchyma with active bleeding. A significant subcapsular splenic hematoma is also noted.

A 19-year-old healthy male was referred to Our Institution following severe blunt abdominal trauma sustained in a motorcycle-truck collision. He was transported to the Emergency Department within one hour of the trauma and was hemodynamically stable on arrival. Nonetheless, the CT scan revealed multiple high-grade injuries, the most severe being an AAST Grade V hepatic lesion (Figure 1), consisting of a deep central laceration extending through the segments IV and VIII with active bleeding, avulsion of the left hepatic vein, and marked hypoperfusion of segment III. Additional findings included aneurysmal dilatation of the left portal vein, complete avulsion of the right renal artery (AAST Grade V), a Grade III splenic laceration with a large subcapsular hematoma, and a 2 cm duodenal hematoma (AAST Grade II) involving the first and second portions. No significant lesions were identified to the brain, chest, or extremities, and the patient was classified with an Injury Severity Score (ISS) of 25. Immediately after the CT scan, the patient's clinical condition worsened, with the onset of hypotension, prompting emergent surgical intervention. Acute care surgeons initially performed routine four-quadrant abdominal packing, which temporarily restored hemodynamic stability and allowed identification of the major bleeding sources. Massive hepatic hemorrhage was immediately identified, and a temporary Pringle maneuver was applied to obtain vascular inflow control. Nevertheless, persistent hemodynamic instability due to ongoing splenic hemorrhage and a large right-sided retroperitoneal hematoma required splenectomy and right nephrectomy to achieve sufficient physiological stabilization with permissive hypotension, thereby allowing intraoperative assessment by the hepatobiliary and liver transplant team, which is available at our centre. A complete transection of the right hepatic duct and total avulsion of the left hepatic vein were promptly identified. Despite prior damage-control maneuvers, the extent of bilobar parenchymal disruption precluded definitive hemostasis. In addition to persistent hemorrhage, the combined major vascular and biliary injuries were judged to predict inevitable progression to acute liver failure (ALF), and LT was deemed the only viable life-saving option. The patient was immediately listed for transplantation as a national emergency. By coincidence, an ABO-compatible liver was available on site from an ongoing deceased-donor procurement, prompting a shift allocation to the emergency LT. A total hepatectomy was therefore performed, thereafter a temporary portocaval shunt was created as a bridge to LT while awaiting completion of donor organ procurement. The portocaval shunt allowed portal venous inflow to be diverted into the inferior vena cava, which was then decompressed via a veno-venous bypass established between the left great saphenous and left axillary veins, ensuring effective extracorporeal circulation and preventing splanchnic and systemic venous congestion. LT was subsequently carried out using the conventional caval replacement technique. Vascular anastomoses were constructed in an end-to-end fashion, while biliary drainage was temporarily diverted externally due to concern over duodenal integrity. The overall duration of the anhepatic phase was 90 min, whereas the temporary portocaval shunt remained *in situ* for 60 min.

**Figure 2:**
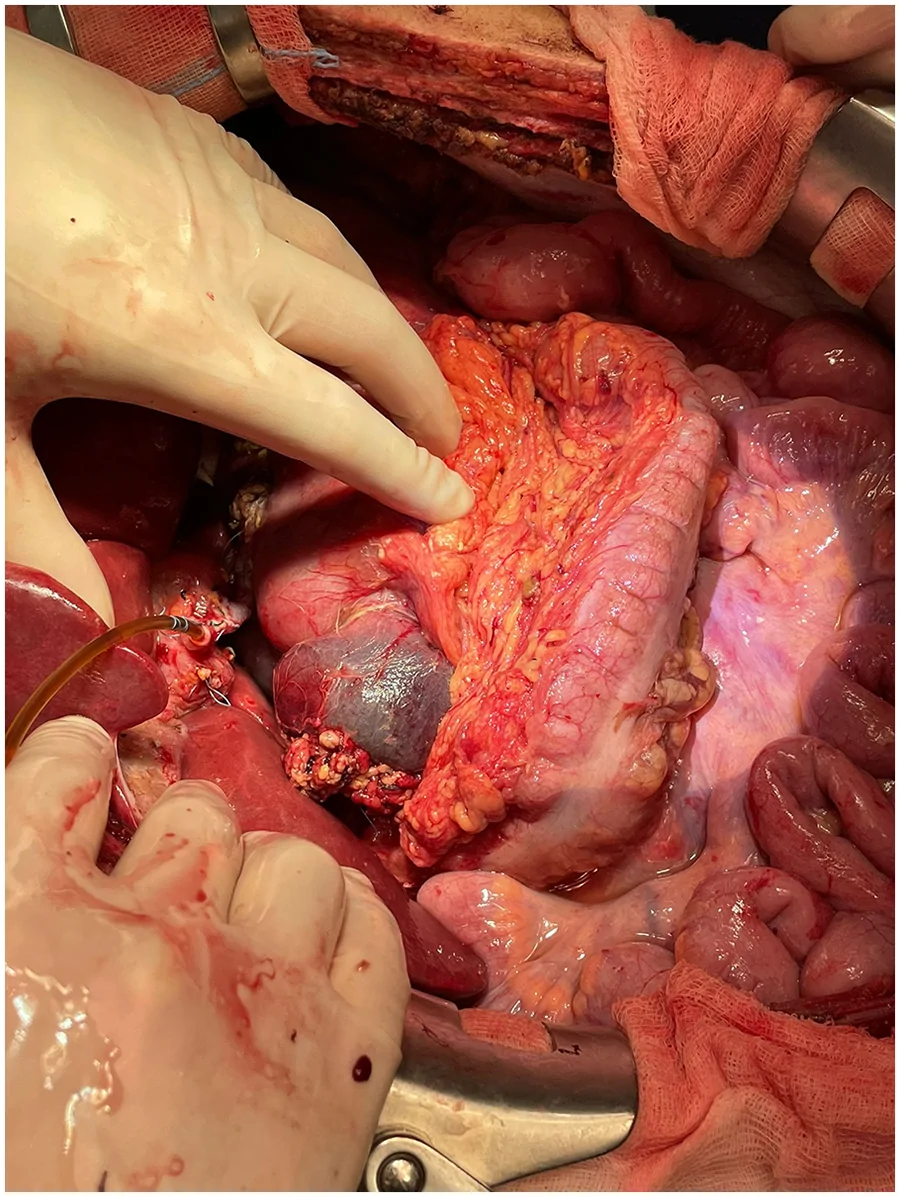
Intraoperative view during the 48-hour second-look procedure following liver transplantation, demonstrating extension of the hematoma involving the second portion of the duodenum. The externally drained biliary duct is visible on the left.

**Figure 3:**
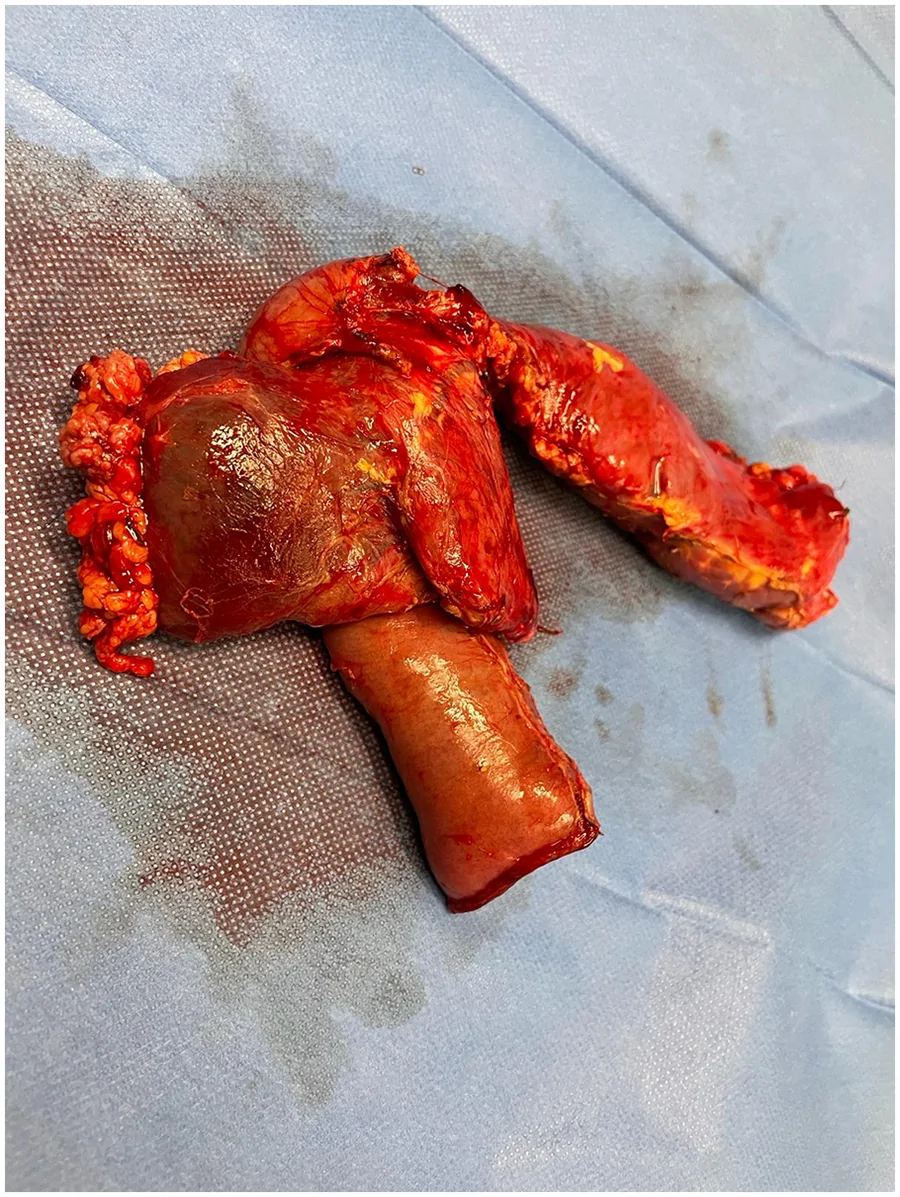
Total pancreatectomy performed during post-transplant re-exploration. An extensive duodenal hematoma is clearly identifiable.

**Figure 4:**
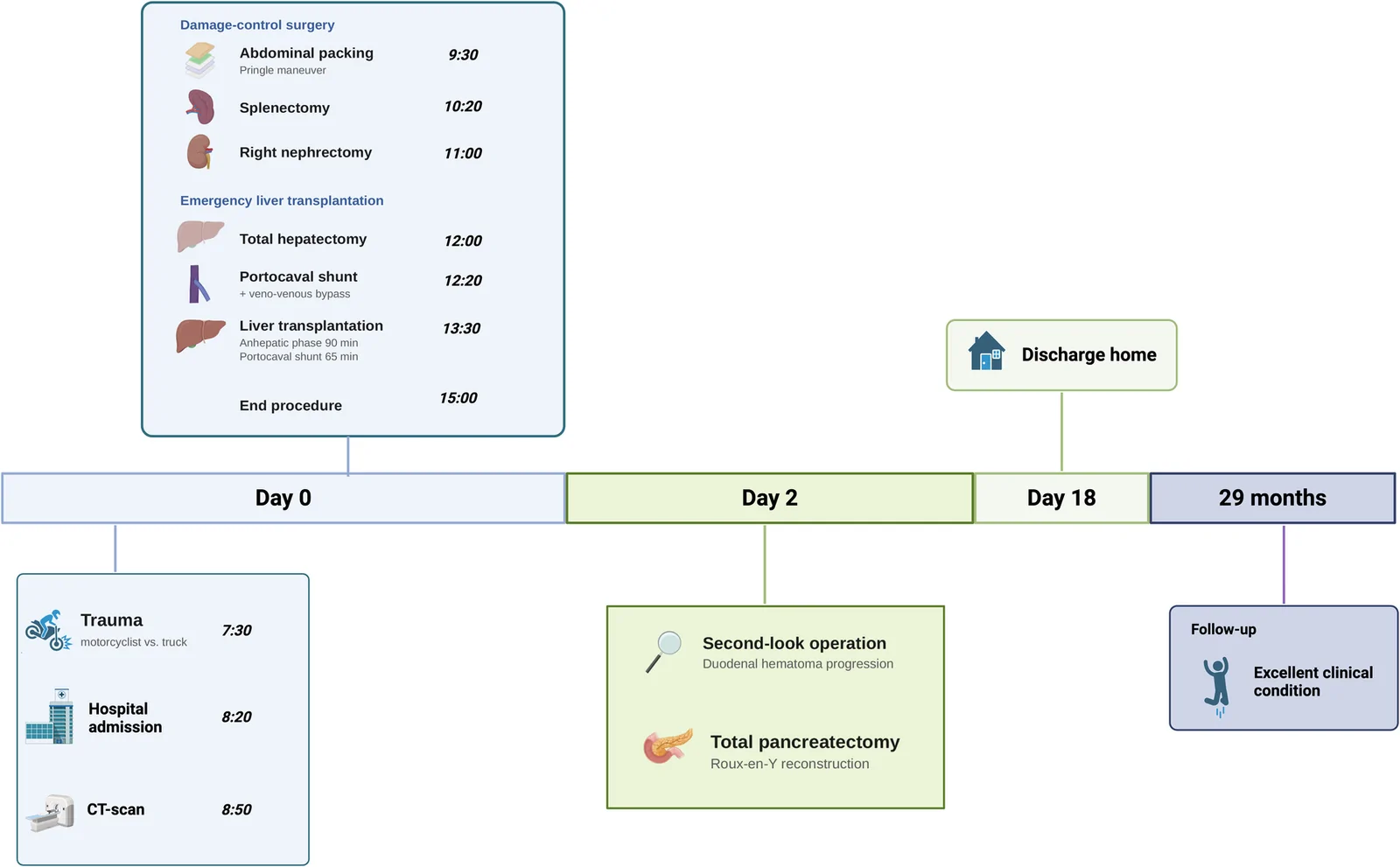
Timeline of the clinical course.

Gastroduodenal artery (GDA) ligation was performed, and transparietal reinforcing sutures were placed on the duodenal wall to address the concerning hematoma and reinforce tissue integrity. Ultimately, a temporary abdominal closure was achieved using a prosthesis as a bridging technique to prevent fascial retraction, with a second-look scheduled 48 h later. During abdominal re-exploration, the duodenal hematoma had markedly enlarged, and the duodenal wall appeared at imminent risk of rupture as shown in Figure 2. Nonetheless, performing a pancreatoduodenectomy just two days after LT would have jeopardized vascular anastomoses in the event of a pancreatic fistula. Thus, a total pancreatectomy was performed (Figure 3) with a Roux-en-Y reconstruction, and primary abdominal wall closure was achieved. Immunosuppressive therapy was initiated with basiliximab (POD 0 and 4 after LT) and steroids, and then maintained with tacrolimus and mycophenolic acid. The postoperative course was uneventful, and the patient was discharged 18 days after LT. The patient received appropriate post-splenectomy vaccinations and was started on insulin replacement therapy. At last follow-up, 29 months after transplantation, he remained in excellent clinical condition, apart from difficult glycemic control, for which pancreatic islet transplantation is currently being considered, while renal function remained within normal limits. The timeline of the clinical case is presented in Figure 4.

## Discussion

Liver transplantation (LT) is an exceptional salvage strategy in hepatic trauma, accounting for fewer than 0.2% of cases and approximately 0.04% of all liver transplants (8). Careful assessment of the patient's physiological status, associated extrahepatic injuries, and potential contraindications is essential, as brain death remains the only absolute contraindication, while severe concomitant injury to other vital organs necessitates multidisciplinary evaluation. Our case report supports the feasibility and non-futility of LT combined with multivisceral resection after a severe polytrauma. To the best of our knowledge, no previous reports in the literature have described emergent LT performed in combination with splenectomy, nephrectomy, and total pancreatectomy.

Prompt surgical management, guided by DCS principles and led by the acute care surgeons, proved crucial during the resuscitative phase. Splenectomy and right nephrectomy were subsequently performed as key bridging interventions, achieving temporary hemodynamic stabilization and enabling a precise assessment of hepatic damage. As reported by Barrie and colleagues, the availability of an on-site HPB service is associated with improved survival (9); in our case early involvement of transplant surgeons enabled a timely and accurate assessment of liver injury and prompt evaluation for LT. This case highlights the critical importance of an integrated multidisciplinary approach, whereby early involvement of a specialized team should be considered mandatory in cases of major abdominal trauma with extensive hepatic involvement.

The decision to proceed with a planned second look proved crucial for the early identification of a progressive duodenal hematoma with high risk of duodenal wall rupture, which could have compromised the survival benefit. Similarly, the choice to proceed with a preemptive total pancreatectomy reflects a carefully balanced and nuanced risk-benefit assessment achievable only within a specialized team. During the initial laparotomy, a conservative strategy for the duodenal injury was attempted, involving GDA ligation and placement of transparietal reinforcing sutures, with planned reassessment at 48 h. At second look, however, progressive enlargement of the hematoma rendered organ preservation unfeasible due to an unacceptably high risk of failure, and a total pancreatectomy was therefore performed. This procedure is generally avoided due to the resulting surgical diabetes, particularly in young patients; however, the risk of a clinically relevant postoperative pancreatic fistula (POPF) following a Whipple procedure in this case was considered to outweigh the already high 15% incidence reported in the literature, particularly in the setting of such severe physiological derangement, with the leakage of pancreatic enzymes had the potential to significantly jeopardize adjacent fresh vascular anastomoses (10). Furthermore, although pancreatoduodenectomy has been reported as feasible in selected cases of duodenopancreatic trauma (11), in our case the development of a POPF would have exposed an already highly vulnerable patient—immunosuppressed after transplantation and asplenic—to a substantially increased risk of infected collections, with potentially devastating consequences. Although the indication for total pancreatectomy may be debatable, the absence of postoperative complications and the uneventful follow-up support its appropriateness in this case, with islet cell transplantation representing a novel strategy to potentially mitigate the long-term consequences of surgically induced diabetes.

Key prognostic factors supporting the decision to proceed with emergent LT included the patient's young age, absence of comorbidities, and a favorable balance between the expected survival benefit, perioperative mortality risk, and likelihood of graft loss. Although the patient sustained severe abdominal injuries, the absence of major extra-abdominal trauma, particularly traumatic brain injury, precluded no contraindications to LT or concerns regarding appropriate graft allocation. Moreover, the ISS of 25 was consistent with severe yet potentially survivable trauma and remained below the threshold of 33 proposed as a contraindication to emergency LT (12). In our case, the prompt availability of a transplant unit and the exceptionally timely availability of an ABO-compatible graft was pivotal in achieving a favorable outcome, resulting in a short anhepatic phase of 90 min, which is clinically relevant given that the literature reports an increased risk of graft dysfunction when the anhepatic phase exceeds 100 min, with a corresponding reduction in one-year patient survival (67% vs. 92%) (13).

Our experience supports the safety of a meticulously performed two-stage approach and is consistent with the findings of Krawczyk and colleagues (12). Early hepatectomy allowed control of bleeding and restoration of hemodynamic stability, while also mitigating the systemic injury associated with toxic liver syndrome secondary to cytokine storm in ALF secondary to extensive hepatic necrosis. This approach effectively limited the release of damage-associated molecular patterns (DAMPs), pro-inflammatory cytokines (including TNF*α*, IL-1β, IL-6, IL-8, and CXCL10), and proteolytic degradation products (14). These inflammatory mediators typically trigger a systemic immune response that could damage the remaining kidney, finally leading to multiple organ failure (MOF), while impaired hepatic ammonia metabolism increases the risk of hepatic encephalopathy (15). During the anhepatic phase, a temporary porto-caval shunt exerts a protective effect by preventing splanchnic venous congestion and toxic metabolite accumulation, thereby reducing ischemia-reperfusion injury (IRI) and graft dysfunction, and is associated with lower rates of major postoperative complications and mortality (16). Similarly, systemic veno-venous bypass represents an additional protective strategy that can be employed in selected complex cases, particularly in unstable patients, as it helps maintain hemodynamic stability by enhancing venous return and reducing both splanchnic and renal congestion (17–19). Together, these approaches contribute to improved physiological stability during LT.

According to current international guidelines, most hepatic injuries are managed non-operatively, whereas surgery is generally reserved for hemodynamically unstable patients or in cases of NOM failure (5). Among patients requiring operative management, reported survival rates approach 80%, but decrease to approximately 50% in the presence of high-grade liver injuries (AAST Grades IV-V) (20). However, despite successful hemorrhage control, both surgical and radiological interventions may result in hepatic ischemia which, when combined with extensive parenchymal damage, can precipitate irreversible ALF. This complication may arise during either the early or late post-traumatic period and remains the most frequently reported indication for LT in this setting (12, 21).

In our case, the indication for emergency LT was twofold. First, although DCS followed by splenectomy and nephrectomy achieved temporary hemodynamic stabilization, definitive control of the hepatic bleeding remained unattainable. Second, detailed assessment of the liver injury demonstrated extensive vascular and biliary disruption, rendering progression to ALF inevitable. In this context, emergency LT represented the safest and most effective therapeutic option, a strategy further supported by the expected availability of a suitable ABO-compatible donor within a short timeframe.

As reported in a multicenter study by Orozco et al., post-traumatic LT achieved one- and five-year patient survival rates of 81.8% and 66.5%, and graft survival rates of 71.2% and 57.4%, respectively, without statistically significant differences compared with LT performed for other indications (8). Notably, the five-year patient survival exceeds the 50% threshold commonly used to define futility in transplant surgery (22). Due to its rarity, no established guidelines exist for this clinical scenario. The European Liver Transplant Registry has suggested that LT should be considered in patients with AAST Grade < V injury and ISS <33, when all other therapeutic options have been exhausted and in the absence of concomitant extra-abdominal injuries of Grade ≥ III (12), whereas Kaltenborn et al. reported no significant association between ISS and either patient or graft survival (23). Although the ISS remains a well-established prognostic indicator in trauma care, its predictive utility in the setting of LT following liver trauma has yet to be clearly defined. Overall, patients with severe hepatic trauma (AAST grade V or higher) should be evaluated for LT on a case-by-case basis. The main limitation of this study is its single-case nature, as each is influenced by multiple and heterogeneous clinical factors. Additionally, the absence of an on-site transplant centre in many institutions may significantly affect outcomes and limit generalizability.

In conclusion, massive hepatic disruption requires early centralization and referral to a transplant centre after initial stabilization. The decision to proceed with LT must be individualized and based on a comprehensive assessment of multiple clinical factors. When NOM fails, DCS remains crucial to restore physiological stability and allow appropriate HPB assessment and timely evaluation for transplantation. This case highlights that, in carefully selected patients, an aggressive yet highly individualized surgical strategy may be justified and associated with meaningful survival benefit, thereby supporting the potential role and non-futility of LT even in such exceptional and complex scenarios.

## Patient's perspective

He demonstrated a clear understanding of the complexity of his clinical condition and the challenges associated with its management. Throughout follow-up, he expressed profound gratitude toward the multidisciplinary team involved in his care, acknowledging their expertise, dedication, and coordinated approach. He also conveyed sincere appreciation to the organ donor and the donor's family for their invaluable gift. At the latest follow-up assessment, the patient described complete reintegration into normal daily activities and social functioning.

## Data Availability

The original contributions presented in the study are included in the article/Supplementary Material, further inquiries can be directed to the corresponding author.
